# ASM LabCap’s contributions to disease surveillance and the International Health Regulations (2005)

**DOI:** 10.1186/1471-2458-10-S1-S7

**Published:** 2010-12-03

**Authors:** Steven Specter, Lily Schuermann, Celestin Hakiruwizera, Mah-Séré Keita Sow

**Affiliations:** 1 University of South Florida Medical Center, Tampa, FL, USA; 2 American Society for Microbiology, Washington, DC, USA

## Abstract

The revised International Health Regulations [IHR(2005)], which requires the Member States of the World Health Organization (WHO) to develop core capacities to detect, assess, report, and respond to public health threats, is bringing new challenges for national and international surveillance systems. As more countries move toward implementation and/or strengthening of their infectious disease surveillance programs, the strengthening of clinical microbiology laboratories becomes increasingly important because they serve as the first line responders to detect new and emerging microbial threats, re-emerging infectious diseases, the spread of antibiotic resistance, and the possibility of bioterrorism. In fact, IHR(2005) Core Capacity #8, “Laboratory”, requires that laboratory services be a part of every phase of alert and response.

Public health laboratories in many resource-constrained countries require financial and technical assistance to build their capacity. In recognition of this, in 2006, the American Society for Microbiology (ASM) established an International Laboratory Capacity Building Program, LabCap, housed under the ASM International Board. ASM LabCap utilizes ASM’s vast resources and its membership’s expertise—40,000 microbiologists worldwide—to strengthen clinical and public health laboratory systems in low and low-middle income countries. ASM LabCap’s program activities align with HR(2005) by building the capability of resource-constrained countries to develop quality-assured, laboratory-based information which is critical to disease surveillance and the rapid detection of disease outbreaks, whether they stem from natural, deliberate or accidental causes.

ASM LabCap helps build laboratory capacity under a cooperative agreement with the U.S. Centers for Disease Control and Prevention (CDC) and under a sub-contract with the Program for Appropriate Technology in Health (PATH) funded by the United States Agency for International Development (USAID). Successful activities of ASM LabCap have occurred throughout Africa, Asia, Central America and the Caribbean. In addition, ASM LabCap coordinates efforts with international agencies such as the WHO in order to maximize resources and ensure a unified response, with the intended goal to help build integrated disease surveillance and response capabilities worldwide in compliance with HR(2005)’s requirements.

## Background

### IHR(2005)

In 2005, the World Health Assembly (WHA) adopted the revised International Health Regulations [IHR(2005)], which require 194 States Parties, including all the Member States of the World Health Organization (WHO), to develop core capacities to detect, assess, report, and respond to public health threats [1]. The WHO is working with public and private sector partners around the world to help countries achieve IHR-mandated core capacities by 2012.

### ASM LabCap Program

In 2005, the U.S. Centers for Disease Control and Prevention (CDC), specifically the Division of Global HIV/AIDS (DGHA), formerly the Global AIDS Program (GAP), approached the American Society for Micro biology (ASM) for assistance with capacity-building of global HIV and clinical microbiology laboratories in resource-constrained countries. The CDC and ASM have since entered into two cooperative agreements (2005 and 2009), and ASM established an International Laboratory Capacity Building Program, LabCap, housed under the ASM International Board. The main mission of ASM LabCap is to utilize ASM’s vast resources and its member ship expertise — 40,000 microbiologists world wide — to strengthen clinical and public health laboratory systems in low and low-middle income countries, primarily through the training and onsite mentoring of laboratory staff . The program began in 2006 as a result of the first CDC/DGHA-ASM cooperative agreement.

ASM is well-equipped to play a major role in the global fight against infectious diseases. More than 5,000 ASM members are clinical microbiologists, many with years of experience developing laboratory quality assurance (QA)/quality control (QC) programs, managing laboratories, instituting good laboratory practices, providing training for performance of laboratory duties, and designing diagnostic tools and procedures. ASM LabCap rapidly engages these experts from around the world to develop and roll-out laboratory training and mentoring programs. These successful activities have to-date occurred in 17 countries: Botswana, China, Côte d’Ivoire, Guatemala, Guyana, Haiti, India, Kenya, Mozambique, Namibia, Nigeria, Rwanda, Tanzania, Thailand, Vietnam, Zambia, and Zimbabwe.

As more countries move toward implementation of infectious disease surveillance programs, such as required by IHR(2005), the strengthening of clinical microbiology laboratories becomes increasingly important because they serve as the first line responders to detect new and emerging microbial threats, re-emerging infectious diseases, the spread of antibiotic resistance, and the possibility of bioterrorism. In fact, IHR(2005) Core Capacity #8, “Laboratory”, requires that laboratory services be a part of every phase of alert and response, including detection, investigation and response, with laboratory analysis of samples performed either domestically or through collaborating centers. States Parties thus need to establish mechanisms for providing reliable and timely laboratory identification of infectious agents and other hazards likely to cause public health emergencies of national and international concern [2].

Although not specifically designed in the context of IHR(2005), ASM LabCap’s program activities align with this effort by building the capability of resource-constrained countries to develop quality-assured, laboratory-based information which is critical to disease surveillance and the rapid detection of disease outbreaks, whether they stem from natural, deliberate or accidental causes. In the elaboration of its programs, ASM LabCap co-ordinates efforts with international and U.S. government (USG) agencies and non-profit organizations such as the WHO, the U.S. Agency for International Development (USAID), divisions of the CDC, and other international organizations in order to maximize resources and ensure a unified response.

ASM LabCap’s various programs directly contribute to global laboratory capacity building efforts. These activities include:

• U.S. President’s Emergency Plan for AIDS Relief (PEPFAR) initiatives

• ASM LabCap-CDC training cooperation

• International Emerging Infections Program (IEIP) initiatives

• ASM - Program for Appropriate Technology in Health (PATH) collaboration in India

• Tuberculosis (TB) Indefinite Quantity Contract (IQC) partnership with PATH, USAID

## ASM LabCap programs and activities

### PEPFAR initiatives

Under a renewed five-year cooperative agreement with the CDC, ASM LabCap is collaborating with CDC/DGHA on capacity building of global HIV and clinical microbiology laboratories in resource-constrained countries, including Botswana, Côte d’Ivoire, Ethiopia, Guyana, Haiti, Kenya, Mozambique, Namibia, Nigeria, Rwanda, Tanzania, Vietnam, and Zambia, in support of CDC’s PEPFAR initiatives. Additional countries/regions that ASM LabCap will be supporting in 2011 via this agreement include the Democratic Republic of Congo, Central Asian Republics, and Ukraine.

Under the direction of the U.S. Global AIDS Coordinator’s Office, CDC/DGHA is a partner in the unified USG effort to implement PEPFAR. The CDC’s longstanding partnerships with ministries of health around the world support improvement of critical infrastructure and services to build sustainable national public health capacity and help resource-constrained countries prevent HIV infection and improve treatment, care, and support for people living with HIV/AIDS. ASM LabCap’s partnership with CDC/DGHA has focused primarily on laboratory diagnostic strengthening for TB and other HIV/AIDS-related opportunistic infections (OIs). ASM LabCap staff and consultants travel to countries to provide onsite technical assistance and mentoring in an effort to help establish and fully transfer quality-assured laboratory diagnostic capacity to laboratorians. Ongoing activities include:

• Assessing laboratory systems and laboratory capacity mapping

• Mentoring and training of laboratory staff

• Providing technical support for the customization and rollout of consensus training packages and programs

• Assisting with the strengthening of laboratory quality systems, including development of QC procedures, QA programs, and standard operating procedures (SOPs)

• Providing guidance for the standardization and selection of equipment and reagents

• Establishing, optimizing, and validating laboratory techniques and procedures

• Assisting with the establishment of national public health/reference laboratories

• Supporting the development of specimen referral networks

• Assisting with the coordination of infectious disease surveillance and outbreak response

• Developing national strategic plans for public health laboratory networks

• Guiding the development or optimization of national laboratory policies

• Assisting laboratories in their preparations for accreditation

• Developing a certification program for laboratory personnel

Key elements of the IHR(2005) laboratory core capacities are laboratory capacity mapping, specimen collection, hand ling, and transport, biosafety, laboratory-based investigation, QA, and reporting/communications [3]. ASM LabCap activities, as outlined above, are consistent with these except for laboratory-based investigation. Note Table 1, which outlines select ASM LabCap country activities.

**Table 1:** ASM LabCap CDC/PEPFAR select program country activities

	Botswana	Côte d’Ivoire	Kenya	Nigeria
Laboratory renovations		Working with 3 TB labs, including NTRL to assist with laboratory renovation design plans to implement TB culture capacity.	Offering ongoing technical guidance on the renovations of 2 national-level reference labs, which includes BSL-3 capacity, as well as provincial & district level labs	Offered TA to renovate 2 national-level & 5 regional labs for implementation of new TB diagnostics.
Procurement of equipment & supplies		Assisted NRL to develop lists of equipment, reagent and supply needs for three national TB laboratories seeking to implement TB culture capacity.	Worked with Kenyan stakeholders to identify & provide a list of required equipment and supplies for the central & provincial level microbiology labs	
National-level training workshops	Worked with national team to customize the consensus *AFB Smear Microscopy Training Package for Botswana* & prepare a plan to roll out the training to lab workers nationally.	Together with CDC & national TB TWG, customized & rolled-out ASM-translated ACILT French TB culture & DST course at NTRL.	With the Central Microbiology Reference Laboratory (CMRL), conducted basic microbiology workshops for Provincial and District Level-5 laboratory technologists	Assisted with facilitating national-level molecular biology training events to reinforce new diagnostic methods.
National EQA schemes	Conducted a pre-implementation assessment of Botswana’s existing TB and Bacteriology EQA program then worked with the national EQA Unit and National Health Laboratory to develop a comprehensive expansion plan & the supporting documentation (forms, procedures, and electronic databases).	Offered TA to NTCP to help implement blinded rechecking of sputum smears & strengthen supervisory visit methodology for the national TB microscopy EQA program.		Offered TA to assist establishment of national EQA schemes to monitor quality, initially as related to a national drug resistance survey.
Establishment/ expansion of National Reference Laboratory	Assisted with the improvement of antimicrobial resistance surveillance.	Working with NRL to redefine core functions for microbiology, STIs & TB.	Working with NPHLS & CDC-Kenya to define & establish the core functions of the CMRL.	
SOPs	Technical guidance offered review & revision of national SOPs, including standardizing procedures & forms for the national QA program & AST.		Worked with NPHLS & CMRL staff to identify essential tests for the CMRL & develop the corresponding SOPs; developing national microbiology SOPs.	Reviewed & recommended updates to 31 technical SOPs for TB to be used as nationally-approved standardized SOPs.
National Strategic Planning & Policy Development	Working with NHL’s Microbiology Laboratory to expand services and strengthen microbiology services nationally. Assisted with formulating a strategic plan to upgrade the NHL’s capacity & transfer routine testing to a new hospital lab.	Participated in MOH-driven National Strategic Planning Workshop for TB, HIV and Malaria labs & offered recommendations for integrating lab services, as well as assisting with guiding elaboration of key policy documents.		
WHO-AFRO & international accreditation		Assisting, via onsite mentoring, 2 national-level labs with achieving WHO-AFRO accreditation status, using ASM-translated disease specific & lab management training modules.	Mentoring the CMRL and provincial microbiology labs to achieve WHO/AFRO accreditation goals.	Assisting, via onsite mentoring & technical guidance, 3 national-level labs with incorporating QMS principles toward achieving WHO-AFRO accreditation status.

### ASM LabCap-CDC training cooperation

ASM LabCap has assisted CDC with developing and rolling out training events for laboratory personnel capacity building. These events include two international courses on Acid-fast bacilli (AFB) smear microscopy external quality assessment (EQA), one in English in Tanzania and one in French in Senegal; a national French-language *Mycobacterium tuberculosis* (Mtb) culture, identification, and drug susceptibility testing (DST) in Côte d’Ivoire; seven national basic microbiology workshops in Botswana, Kenya, Mozambique, Tanzania, and Zambia; and a national workshop for laboratory support of enteric disease outbreak surveillance and response in Kenya. A brief description of these programs follows.

#### AFB Smear Microscopy EQA workshops – Tanzania and Senegal

ASM LabCap facilitated two five-day workshops entitled “Strengthening AFB Smear Microscopy EQA Programs”, one in English in December 2009 in Dar es Salaam with participants from Angola, Bahamas, Barbados, Botswana, Ethiopia, Jamaica, Kenya, Lesotho, Malawi, Mozambique, Namibia, Rwanda, Swaziland, Tanzania, Vietnam, Zambia, and Zimbabwe, and a francophone version in August 2010 in Dakar with participants from Senegal, Cameroon, Haiti, and Côte d’Ivoire. In line with Article 44 of the IHR(2005), which calls for States to share technical cooperation and assistance in order to strengthen and maintain public health capacities, these platforms allowed the participating countries to network and share experiences, challenges, best practices, and lessons learned from EQA implementation, as well as assess new opportunities to integrate AFB smear microscopy into other laboratory disciplines. ASM LabCap is providing follow-up support to many of the participating countries.

#### French-language Liquid Culture and DST course

From February 22 through March 5, 2010, ASM LabCap and CDC/PEPFAR-Côte d’Ivoire organized in Abidjan, Côte d’Ivoire, its first course on Mtb culture, identification and DST for French-speaking African laboratorians. Scientists, managers, and technicians from the National TB Program and four major Ivorian TB laboratories attended the two-week course held at the National TB Reference Laboratory housed in the Pasteur Institute of Côte d’Ivoire (IPCI). The course was the first national training event in Côte d’Ivoire to help increase detection and reporting of multi-drug resistant (MDR)-TB using newer, more rapid technology. TB, in its MDR and extensively drug resistant (XDR) forms, is a disease with the potential of causing public health emergencies which fall within the scope of IHR(2005). Its early detection, control, and containment in endemic regions of Africa, such as Côte d’Ivoire, are steps towards improving international public health security.

#### Basic clinical microbiology course – Botswana, Kenya, Mozambique, Tanzania, and Zambia

ASM consultants have facilitated workshops on basic bench top procedures for clinical microbiology for a total of 145 participants thus far. The goal of each workshop, as developed by an ASM LabCap Committee Member, was to enable clinical microbiology laboratory technologists to instruct clinicians on proper choice and collection of patient samples for microbiological studies, to properly handle the samples in the laboratory with respect to initial testing and culture processes, to adequately identify the major pathogens in each site (and to not report non-pathogens), and to perform necessary antimicrobial susceptibility tests (AST) on those pathogens. These procedures are encompassed in a series of flow-charts and are demonstrated with real cultures and organisms. Participants learned in the laboratory by performing the procedures themselves. A list of the reagents and simple supplies used in the laboratory were provided to the participants for training and QC purposes in their own facilities. ASM LabCap places consultants in-country to work with the various participating laboratories as a follow-up to the training.

Course topics covered during these workshops relevant to the IHR(2005) included procedures for AST, detection of *Vibrio cholerae*, and other pathogen-causing epidemic diarrheal diseases, meningococcal meningitis, and respiratory infections.

#### CDC-ASM Joint Workshop for Laboratory Support of Enteric Diseases Outbreak Surveillance and Response – Kenya

Since 2008, there have been ongoing diarrheal outbreaks in Kenya that have spread to several districts throughout the country, including Nairobi. Performing accurate and timely laboratory identification of the etiological agents of outbreaks has been challenging for Kenya’s District and Provincial hospitals, which have limited skills and resources. Consistent with IHR(2005)’s focus on strengthening laboratory-based surveillance systems for epidemic-prone diseases such as cholera, in October 2009, 39 district-based laboratory technologists, surveillance officers, and clinical officers convened in Nairobi for a workshop designed to build their capacity to provide support in enteric disease outbreak surveillance and response. This effort was a collaborative endeavor between CDC/GDD (Global Disease Detection) Kenya Field Epidemiology and Laboratory Training Program (FELTP), the Kenyan Ministry of Public Health and Sanitation (MoPHS), the CDC National Center for Emerging, Zoonotic & Infectious Diseases (NCEZID) in Atlanta, CDC/DGHA, and ASM LabCap. The course illustrated the critical link between epidemiology, outbreak investigation, and both rapid and traditional detection by the laboratory.

The trainees were presented with the basic principles of epidemiology and outbreak detection; determination of proper samples to collect in an outbreak; collection, packaging, and transport of biological samples from an outbreak; and rapid testing for detection of *Vibrio cholerae, Shigella dysenteriae* Type 1, *Salmonella enterica* serovar *typhi*, and Shiga toxin-producing *E. coli* from biological samples. Furthermore, ASM LabCap facilitators discussed essential microbiological procedures and techniques for processing samples and traditional methods for culture, identification and susceptibility testing of the four enteric pathogens. Overviews of Gram staining, laboratory diagnosis of common HIV-related OIs and other outbreak pathogens, QC, and media preparation were also included.

### International Emerging Infections Program (IEIP) initiatives

IEIP is an international effort by the CDC as part of its Global Disease Detection Program to built capacity around the world to detect emerging infectious disease. ASM LabCap is providing technical expertise and consultation for building laboratory capacity and improving clinical microbiology for the diagnosis of respiratory diseases in select IEIP sites. Each site is set to implement active, population-based surveillance for pneumonia with varying levels of microbiological capacity. Below are descriptions of the IEIP sites that ASM LabCap has supported to-date, and the specific support provided by ASM.

#### China

The U.S. CDC and China’s CDC collaborate to conduct active surveillance for patients hospitalized with community-acquired pneumonia in China. A three-month pilot project evaluating procedures for patient enrollment and specimen collection, transport, testing, and reporting, was conducted from mid-November 2008 to February 2009 in three sites (Panjin, Liaoning Province; Jingzhou, Hubei Province; Zhuhai, Guangdong Province). Patients enrolled in this study had specimens collected for microscopic examination, bacterial culture, viral culture, urine antigen testing, and polymerase chain reaction (PCR)-based identification. During the pilot, ASM LabCap evaluated the capacity at each site to perform non-PCR based testing, to provide guidance regarding necessary supplies and equipment at each site, and to write SOPs. ASM LabCap also provided additional technical guidance for the finalization of the study protocol.

#### Guatemala

ASM LabCap worked with the CDC-Guatemala office for Central America and Panama and the Guatemalan Ministry of Health to provide technical expertise and consultation for improving the diagnosis of respiratory diseases in Guatemala. ASM LabCap visited labs in Guatemala City and Cuilapa and reviewed blood culture processing and provided recommendations for improving species identification, particularly *Streptococcus pneumoniae*, using the available BacT/Alert automated blood culture system. ASM LabCap additionally offered guidance for optimizing susceptibility testing via disk diffusion.

#### Thailand

The Thai provinces of Nakhon Phanom and Sa Kaeo are part of an active-population based surveillance system for community-acquired pneumonia under a Thai Ministry of Public Health (MOPH) - U.S. CDC collaboration. Each has a major hospital with additional small hospitals and clinics. ASM LabCap evaluated procedures for specimen collection, transport, processing, culture, and identification primarily for the detection of *Streptococcus pneumoniae* and provided recommendations to improve and refine their procedures.

### ASM-PATH collaboration in India

The Government of India has allocated substantial funding for developing integrated disease surveillance, with laboratory capacity building as an essential component and TB as a targeted disease for improved surveillance. Since November 2008, ASM LabCap has been working with PATH, on a USAID-funded initiative, to strengthen the capacity of the Intermediate Reference Laboratory (IRL) network to perform Mtb culture and DST (expected to expand to one IRL/state or territory or over thirty laboratories). The ultimate goal of this initiative is to ensure that selected IRLs obtain national accreditation, i.e., be deemed fully proficient in Mtb culture and DST by an overseeing National Reference Laboratory (NRL). States (or territories) with accredited IRLs will then be able to conduct drug resistance surveys (DRS) and implement the DOTS-Plus strategy to treat MDR-TB.

Activities performed by ASM LabCap thus far include:

• An in-depth onsite evaluation of IRLs in 8 states – Orissa, Haryana, West Bengal, Jarkhand, Uttarakhand, Uttar Pradesh, Chhattisgarh and Tamil Nadu – using a new comprehensive, customized IRL assessment tool. The ASM LabCap consultants’ reports constituted the basis for development of accreditation work plans.

• Development of guidelines for preventive maintenance and troubleshooting for equipment at IRLs, and a Biosafety Manual.

• Offering recommendations for the organization of an NRL/IRL Workshop held in November 2009. It was the first time that microbiologists from IRLs had the opportunity to come together and share their experiences in the presence of their NRL supervisors and peers. The workshop was facilitated by, among others, the ASM LabCap consultant, who guided the technical discussions; it was so successful that it has now been decided to hold such a workshop twice yearly.

### TB Indefinite Quantity Contract (IQC) partnership with PATH, USAID

ASM LabCap is also engaged in efforts to control wide scale infectious disease, such as TB. In 2009, through USAID’s TB IQC, ASM partnered again with PATH in a consortium that also includes the Foundation for Innovative New Diagnostics (FIND), Partners in Health, University of California-San Francisco (UCSF), Management Sciences for Health (MSH), Brigham and Women’s Hospital, and Initiatives, Inc. The purpose of the program is to provide extensive support to USAID operating units in the implementation of their TB control and prevention programs through the introduction and expansion of the components of the WHO-recommended STOP TB Strategy. The TB IQC places special emphasis on MDR-TB prevention and control and strengthening TB laboratory capacity.

## Conclusion

The IHR(2005) core capacity laboratory requirements are bringing new challenges for national laboratory systems; these requirements include improved diagnostic capacity, better connectivity intersectorally and internationally, and better quality of laboratory data and reporting. Public health diagnostic laboratories in many resource-constrained countries lack laboratory involvement in disease surveillance, advocacy or resources, supporting infrastructures, biosafety awareness and practices, and pathogen security practices [3]. These countries require financial and technical assistance to build their diagnostic laboratory capacity.

ASM LabCap, through partnerships with the WHO, CDC, and USAID, among others, has been assisting various WHO States Parties with acquiring needed laboratory diagnostic capacity, both as related to human resource and infrastructural developments. These activities are providing resource-constrained countries with the necessary tools to improve their public health laboratory infrastructure, which will enable them to move towards compliance with IHR(2005) requirements. The scope of ASM LabCap’s work enables indigenous laboratorians to more rapidly and effectively identify and respond to a broad spectrum of diseases of public health relevance. The activities are designed around creating sustainability and primarily focused on human resource development, specifically involving local entities in designing and customizing laboratory training tools that address their particular needs while still meeting international standards.

Through the achievements of programs such as ASM LabCap, there is an increased visibility of the critical role laboratories play in health systems, helping advocacy efforts and garnering additional resources for laboratory capacity building efforts, as well as promoting the full integration and participation of laboratorians in the planning and implementation of national and international disease surveillance systems. Furthermore, ASM LabCap’s targeted training and mentoring programs are transferring quality-assured laboratory skills to laboratorians with outcomes that indicate that the quality of the laboratory data are greatly improving, including data recording and reporting mechanisms. ASM LabCap is implementing in each of the supported laboratories a quality management system (QMS) approach that emphasizes the use of SOPs, guidance documents, QA/ QC procedures, biosafety practices and participation in successful EQA schemes; moving the laboratories toward international accreditation is a key activity of ASM LabCap.

In 2006, the WHA made a resolution relative to the immediate and voluntary compliance with IHR(2005); it included a request to “expand and accelerate training efforts in the areas of…laboratory capacity, including regional networking of laboratories, biosafety, and quality control…”, and in 2008, the WHA resolved to pay close attention to the sub-Saharan African laboratory network [4]. ASM LabCap is actualizing an accelerated response to deficiencies in laboratory capacity in many sub-Saharan African countries by helping the countries define and implement laboratory policies and guidance documents, map their public health laboratory capacity, develop human resources capable of implementing IHR(2005) laboratory core capacity requirements, offer laboratory services to test for priority health threats, institute laboratory biosafety and biosecurity practices, and network on a regional and international level, in support of Article 44 of IHR(2005), and expressed in the 2006 and 2008 WHA resolutions.

Meeting IHR(2005) requirements by 2012 will require a great deal of partner collaborations and coordination. The USG is increasing its engagement in IHR(2005) capacity-building. As ASM LabCap is currently primarily supported by USG agencies as a technical arm for assisting low and low-middle income countries with improving their laboratory disease detection capacity, it is anticipated that future projects will be more directly conceptualized in the context of IHR(2005). In addition, ASM LabCap will continue its active collaboration with the WHO and other international organizations in order to further coordinate efforts for building integrated disease surveillance and response capabilities worldwide.

## Abbreviations

ACILT: African Centre for Integrated Laboratory Training; AFB: Acid-Fast Bacilli; ASM: American Society for Microbiology; AST: Antimicrobial Susceptibility Testing; BSL-3: Biosafety Level 3; CDC: U.S. Centers for Disease Control and Prevention; CMRL: Central Microbiology Reference Laboratory; DGHA: Division of Global HIV/AIDS; DOTS-Plus Strategy: Directly Observed Therapy: Shortcourse-Plus Strategy; DRS: Drug Resistance Surveys; DST: Drug Susceptibility Testing; EQA: External Quality Assessment; FELTP: Field Epidemiology and Laboratory Training Program; FIND: Foundation for Innovative Diagnostics; GAP: Global AIDS Program; GDD: Global Disease Detection; IHR: International Health Regulations; IEIP: International Emerging Infections Program; IPCI: Institut Pasteur of Côte d’Ivoire; IRL: Intermediate Reference Laboratory; IQC: Indefinite Quantity Contract; LabCap: International Laboratory Capacity Building Program (ASM); MDR-TB: Multi-Drug Resistant Tuberculosis; MOPH: Ministry of Public Health (Thailand); MoPHS: Ministry of Public Health and Sanitation (Kenya); MSH: Management Sciences for Health; Mtb: *Mycobacterium tuberculosis*; NCEZID: National Center for Emerging: Zoonotic & Infectious Diseases; NHL: National Health Laboratory; NPHLS: National Public Health Laboratory Services; NRL: National Reference Laboratory; NTCP: National Tuberculosis Control Program; NTRL: National Tuberculosis Reference Laboratory; OIs: Opportunistic Infections; PATH: Program for Appropriate Technology in Health; PCR: Polymerase Chain Reaction; PEPFAR: United States President’s Emergency Plan for AIDS Relief; QA: Quality Assurance; QC: Quality Control; QMS: Quality Management System; SOPs: Standard Operating Procedures; STIs: Sexually Transmitted Infections; TA: Technical Assistance; TB: Tuberculosis; TWG: Technical Working Group; WHO: World Health Organization; WHO-AFRO: World Health Organization – Regional Office for Africa; UCSF: University of California-San Francisco; USAID: United States Agency for International Development; USG: United States Government; WHA: World Health Assembly; XDR: Extensively Drug Resistant.

## Competing interests

The authors declare that they have no competing interests.

## Authors’ contributions

All authors contributed equally to the paper. All authors read and approved the final manuscript.
